# Variations in T cell transcription factor gene structure and expression associated with the two disease forms of sheep paratuberculosis

**DOI:** 10.1186/s13567-016-0368-3

**Published:** 2016-08-17

**Authors:** Louise Nicol, Hazel Wilkie, Anton Gossner, Craig Watkins, Robert Dalziel, John Hopkins

**Affiliations:** 1 The Roslin Institute & R(D)SVS, University of Edinburgh, Easter Bush, Midlothian, EH25 9RG UK; 2 Moredun Research Institute, International Research Centre, Pentlands Science Park, Penicuik, Midlothian, EH26 0PZ UK

## Abstract

Two different forms of clinical paratuberculosis in sheep are recognised, related to the level of bacterial colonization. Paucibacillary lesions are largely composed of lymphocytes with few bacteria, and multibacillary pathology is characterized by heavily-infected macrophages. Analysis of cytokine transcripts has shown that inflammatory Th1/Th17 T cells are associated with development of paucibacillary pathology and Th2 cytokines are correlated with multibacillary disease. The master regulator T cell transcription factors *TBX21*, *GATA3*, *RORC2* and *RORA* are critical for the development of these T cell subsets. Sequence variations of the transcription factors have also been implicated in the distinct disease forms of human mycobacterial and gastrointestinal inflammatory diseases. Relative RT-qPCR was used to compare expression levels of each transcript variant of the master regulators in the ileo-caecal lymph nodes of uninfected controls and sheep with defined paucibacillary and multibacillary pathology. Low levels of *GATA3* in multibacillary sheep failed to confirm that multibacillary paratuberculosis is caused simply by a Th2 immune response. However, high levels of *TBX21*, *RORC2* and *RORC2v1* highlights the role of Th1 and Th17 activation in paucibacillary disease. Increased *RORAv1* levels in paucibacillary tissue suggests a role for RORα in Th17 development in sheep; while elevated levels of *RORAv4* hints that this variant might inhibit RORα function and depress Th17 development in multibacillary sheep.

**Electronic supplementary material:**

The online version of this article (doi:10.1186/s13567-016-0368-3) contains supplementary material, which is available to authorized users.

## Introduction

Paratuberculosis (Johne’s disease) is a common intestinal disease of ruminants caused by infection with the intracellular bacterium *Mycobacterium avium* subspecies *paratuberculosis* (Map) [1]. As with tuberculosis [2], clinical disease only develops in a minority of infected individuals, and in sheep the disease can be one of two forms. About 70% of the diseased animals develop multibacillary or lepromatous pathology with lesions in the terminal ileum composed of heavily infected macrophages [3, 4], and the remainder develop paucibacillary or tuberculoid pathology with characteristic lymphocytic infiltration, granulomatous inflammation and few bacteria.

The immunology of paratuberculosis in sheep is also similar to that of the tuberculoid and lepromatous forms of tuberculosis and leprosy [5, 6]. Multibacillary pathology is linked with a strong Th2 response with high levels of interleukin (IL)-5 [7, 8] and paucibacillary disease is associated with an inflammatory Th1/Th17 response, characterized by IL-12, IL-17A and IFNγ [7, 9, 10]. However, the immune response associated with the two forms of the disease is not a simple matter of Th1/Th2 discrimination as there seems to be a total T cell dysfunction and loss of homeostasis in animals with multibacillary pathology [11–13], possibly concerned with changes to co-stimulatory and second messenger expression [14, 15].

In common with tuberculosis and leprosy, the epidemiology of paratuberculosis strongly suggests a genetic susceptibility to disease severity and pathological form [16, 17]. Many of the genes associated with severity of human mycobacterial diseases and pathology belong to the pathways that control differential T cell activation [16]. The polarization of T cells into the various CD4+ T cell functional subsets occurs within organized lymphoid tissue and is largely controlled by the master regulator transcription factors that control differential cytokine production including Tbx21 or T-bet (*TBX21*), GATA-3 (*GATA3*), RORγt (*RORC2*) and RORα (*RORA*); [18]. Tbx21 and GATA-3 are the regulators of Th1 and Th2 differentiation respectively; initially by transactivation of relevant cytokine genes and also by reciprocal repression [19, 20]. RORγt is the major transcription factor that regulates Th17 development, and RORα is required for the maximum production of the cytokine IL-17A [21]. GATA-3 and RORα are also important in the development and function of the different subsets of innate lymphoid cells 2 (ILC2), cells associated with mucosal surfaces in mice and important in the initiation of Th2 responses [22].

Map is also implicated in the pathogenesis of Crohn’s disease [14], and these master regulators play important roles in the pathogenesis of this disease and other inflammatory diseases at mucosal sites. Tbx21 expression is implicated in the abnormal expression of IFNγ in Crohn’s disease and GATA-3 is involved in the immunopathology of ulcerative colitis [23, 24]. Th17 cells function largely in the development of inflammatory reactions and several inflammatory conditions are linked with ectopic Th17 T cell activation; therefore RORγt and RORα are also implicated in inflammatory disease [25, 26]. Alternative splicing of exons (or part of an exon or intron) into a transcript introduces variations into many genes [27] and such variations in the transcripts of the human master regulator transcription factors can modify their functions [28–30].

We have recently identified two transcript variants of both sheep *GATA3* and *RORC2*, a single *TBX21* transcript and five variants of *RORA* [31]. In this study we compared the expression of each transcript variant of these four transcription factors, in the ileo-caecal lymph nodes (ICLN) that drain the disease lesions in the terminal ileum adjacent to the ileo-caecal valve, in sheep with define multibacillary or paucibacillary pathology, to test the hypothesis that the pathological form of sheep paratuberculosis is associated with differential expression of individual T cell transcription factor variants.

## Materials and methods

### Animals, disease diagnosis and tissue collection

Animals with clinical disease were outbred female Blackface or Blackface x sheep with naturally-acquired MAP infection (Table 1) from six farms; healthy, uninfected control sheep were all Blackface ewes from a single farm with no history of paratuberculosis. Paratuberculosis pathology in each animal was confirmed at post-mortem by haematoxylin and eosin and Ziehl-Neelsen histopathology of the terminal ileum lesions and mesenteric lymph nodes [7]. PCR for insertion sequence 900 (*IS900*) was used to confirm infection. There were six animals in each of three groups; multibacillary (M), paucibacillary (P) and uninfected controls (C). Tissue was ICLN removed at post-mortem, cut into blocks of ~0.5 g and placed in five volumes of RNAlater (Ambion, UK), then incubated overnight at 4 °C and stored at −80 °C. No animals were euthanized specifically for this study; infected sheep were humanely culled for clinical reasons and uninfected controls were euthanized for reasons unrelated to this study (uninfected controls).

**Table 1:** **Details of paratuberculosis-diseased and control sheep**

Sheep ID	Breed	Origin^a^	Age (years)	SGI^b^	AFB^c^	*IS900* ^d^	Diagnosis^e^
SH.139	Blackface	A	3	5	4	+, +	M
SH.140	Blackface	A	3	7	4	+, +	M
SH.146	Blackface x	B	2.5	5	4	+, +	M
SH.190	Blackface	C	3	5	4	+, +	M
SH.199	Blackface	C	2	6	4	+, +	M
SH.204	Blackface	D	1.5	6	4	+, +	M
SH.107	Blackface x	E	2.5	2.5	0	+, +	P
SH.147	Blackface x	B	2	3	0	+, +	P
SH.155	Blackface	F	3	2	0	+, +	P
SH.160	Blackface x	B	3	2.5	0	+, +	P
SH.188	Blackface x	B	4	4	1	+, +	P
SH.205	Blackface	D	4	2	0	+, +	P
K207	Blackface	G	2.5	0	0	−, −	C
K208	Blackface	G	2.5	0	0	−, −	C
K213	Blackface	G	2.5	0	0	−, −	C
K224	Blackface	G	2.5	0	0	−, −	C
K227	Blackface	G	2.5	0	0	−, −	C
K229	Blackface	G	2.5	0	0	−, −	C

^a^ Source farms.

^b^ SGI (Severity of Granulomatous Inflammation) grading: based on total number of epithelioid macrophages and leukocyte distribution patterns of the terminal ileum [46].

^c^ AFB (Acid Fast Bacteria)—grading: grades 0–2 were defined as paucibacillary; grades 3–4 were defined as multibacillary observed in terminal ileum tissue.

^d^ *IS900* PCR result using each of the two primer sets.

^e^ Diagnosis: based on histopathological observations.

### *Is900* pcr

ICLN was tested for MAP infection by PCR for *IS900*. Two independent primer sets were used [32, 33]; set 1 (for: GTTCGGGGCCGTCGCTTAGG; rev: GCGGGCGGCCAATCTCCTT) and set 2 (for: CTGGCTACCAAACTCCCGA; rev: GAACTCAGCGCCCAGGAT) generated products of 99 bp and 314 bp respectively. Genomic DNA (gDNA) was purified using the Wizard^®^ Genomic DNA Purification Kit (Promega, UK). All reactions used FastStart Taq DNA Polymerase (Roche Diagnostics, UK) following the manufacturer’s instructions, with 500 ng of gDNA and 0.2 μM of each primer. PCRs used a Veriti^®^ Thermal Cycler (Applied Biosystems, UK) with four reactions performed for each primer set at four different annealing temperatures. PCR parameters were: 5 min at 95 °C, then 35 cycles of 15 s at 95 °C, 15 s at 55 °C, 58 °C, 60 °C, or 62 °C, and 30 s at 72 °C, with final elongation of 10 min at 72 °C. PCR products were separated by 2% agarose gel electrophoresis, purified with a MinElute Gel Extraction Kit (Qiagen, UK); cloned using a TOPO TA cloning kit (Invitrogen, UK) and sequenced using the BigDye Terminator v3.1 Cycle Sequencing Kit and 3730 DNA Analyser (Applied Biosystems). The presence of an amplicon, confirmed by sequencing, from either primer set was taken as a positive indication for the presence of MAP; the absence of an amplicon in all PCR reactions was confirmation of absence of MAP infection.

### Quantitative real-time RT-PCR analysis

Total RNA was obtained from ~20 mg tissue using the Ribopure Kit (Ambion, UK) according to the manufacturer’s instructions, DNA was removed by On-column PureLink^®^ DNase I treatment (Ambion). RNA quantity, quality and integrity were determined by NanoDrop ND-1000 spectrophotometry and Agilent 2200 TapeStation system; all had an RNA Integrity Number of >7.4. cDNA was synthesised from 1 μg RNA using SuperScript™ II RT with RNaseOUT (Invitrogen) and oligo-dT_(15)_ primer (Promega, UK), in a 20 μL final volume. Quantitative real-time RT-PCR (RT-qPCR) was performed in 15 μL volumes containing 7.5 μL FastStart Universal SYBR Green Master (Rox) 2× concentration (Roche), 2 μl template cDNA (diluted 1/10−1/40), 1 μL dNTPs (10 mM) primers and nuclease-free water; primer sequences, Tm and product sizes (Additional file 1) have been published [31]. Reactions were prepared using a CAS-1200™ robot and performed on a Rotor-Gene Q (Qiagen). All reactions had an efficiency >95% and R^2^ > 0.98; PCR amplicons were sequenced to confirm specificity. Three biological (RT) replicates per animal were assayed, each in duplicate and duplicate no-template controls were included in all runs.

Relative gene expression levels were calculated in GenEx 5 (MultiD Analyses AB, Sweden) using the comparative 2−(ΔΔ Cq) method and normalized to the geometric mean of *YWHAZ* and *SDHA.* Fold changes were calculated from ΔCq values using GenEx. The difference between the group means for each gene was analysed using Graphpad Prism 6 by one-way ANOVA to determine overall significance, with Tukey’s multiple comparison test within ANOVA to determine significance between groups.

## Results

### Paratuberculosis-diseased and control sheep

The pathological characteristics of the three groups of animals is shown in Table 1. Representative haematoxylin and eosin and Ziehl-Neelsen histopathology of the lymph node is shown in Additional file 2. All infected animals were *IS900* positive with both primer sets; all amplicons were sequenced and confirmed to be 100% identical with *Mycobacterium avium* subsp. *paratuberculosis* insertion sequence IS900 sequence (accession no. S74401.1). All uninfected control sheep were negative for *IS900* PCR using both primer sets under all four PCR conditions.

### Master regulator transcription variants

Cloning and sequencing of transcription factor transcripts from abomasal lymph node identified two variants of *GATA3*, two variants of *RORC2*, five variants of *RORA* and a single *TBX21* transcript [31]. In summary (Additional file 3), the two *GATA3* variants include the full length *GATA3* (LN848231) and *GATA3v1* (LN848232) that has a single codon deletion at the 5′ end of exon 3 (g.806_808delGAA) that encodes glutamic acid at position 260. The two *RORC2* variants include a full length transcript (*RORC2*, LN848233) and a truncated transcript *RORC2v1* (LN848234) with a 36 bp deletion (g.1237_1272del), which encodes GKYGGVELFRAL at position 359–370. The five *RORA* variants (LN848235–LN848239) have different 5′ sequences, *RORAv1*, *v2*, *v3* and *v5* have different translation start sites, and *RORAv2* and *v4* have identical start sites. All five variants have the same ligand-binding domain but *RORAv2* and *v4* do not encode the DNA-binding domain. *RORAv1*, *v3* and *v5* encode different NH_2_ sequences upstream of the DNA-binding domain.

### *TBX21* and *GATA3* expression

**Figure 1:**
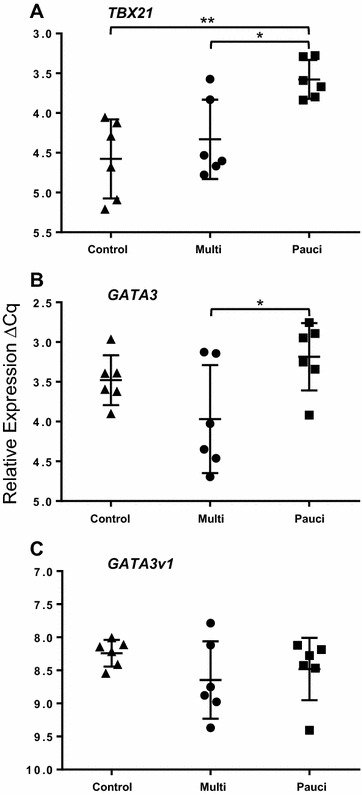
**Relative quantification of** **A** ***TBX21***, **B** ***GATA3*** **and** **C** ***GATA3v1*** **in ICLN of paratuberculosis-diseased and uninfected control sheep.** Each point is the mean ΔCq of three biological replicates per animal, each in duplicate. ANOVA: **A** *TBX21*, *P* = 0.003; **B** *GATA3*, *P* = 0.046; **C** *GATA3v1*, *P* = 0.32; error bars, ± SD. *≤ 0.05, >0.01; **≤0.01, 0.001; ***≤0.001.

Relative RT-qPCR analysis of the master regulator transcription factors that control Th1 and Th2 T cell differentiation showed that there were overall significant differences in the expression of *TBX21* (*P* = 0.003) and *GATA3* (*P* = 0.046) but not the variant *GATA3v1* (*P* = 0.32) (Table 2). Comparison between the groups (Figure 1) showed that *TBX21* was significantly increased in paucibacillary sheep, resulting in a 2.6 fold change in the *P* vs. C comparison (*P* ≤ 0.01) and a −1.88 fold change in the M vs. *P* comparison (*p* ≤ 0.05). Similar analysis for *GATA3* showed a significant −2.08 fold change (*P* ≤ 0.05) in the M vs. *P* comparison, but no significant differences were measured in the M vs. C (−1.6 fold) and *P* vs. C (1.2 fold) comparisons. There were no significant differences between the groups with *GATA3v1* (Figure 1C).

**Table 2:** **Relative expression of transcription factors within the ICLN**

Gene	ANOVA *P* value	Multi vs. control		Pauci vs. control		Multi vs. pauci	
		FC^a^	*P* value	FC	*P* value	FC	*P* value
*TBX21*	*0.003*	1.38	ns	*2.6*	*≤0.01*	*−1.88*	*≤0.05*
*GATA3*	*0.046*	−1.6	ns	1.2	ns	−*2.08*	*≤0.05*
*GATA3v1*	0.32	−1.5	ns	−1.25	ns	−1.2	ns
*RORC2*	*0.0004*	*2.4*	*≤0.05*	*3.48*	*≤0.001*	−1.45	ns
*RORC2v1*	*0.0026*	*3.09*	*≤0.05*	*3.49*	*≤0.01*	−1.12	ns
*RORAv1*	*0.0053*	1.2	ns	*2.49*	*≤0.01*	−*1.9*	*≤0.05*
*RORAv4*	*0.0002*	*2.78*	*≤0.001*	*2.89*	*≤0.001*	−1.04	ns
*RORAv5*	0.07	1.8	ns	2.67	ns	−1.47	ns

^a^ Fold change (ΔΔCq values).

Italics: *P* ≤ 0.05.

### *RORC2* expression

**Figure 2:**
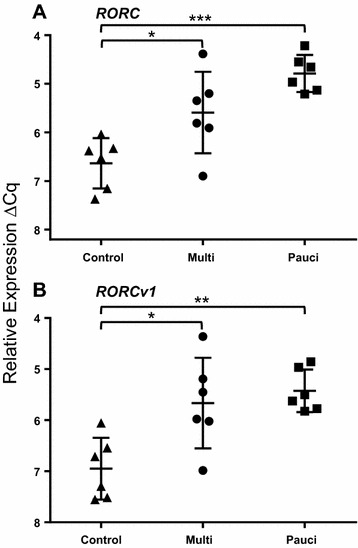
**Relative quantification of** **A** ***RORC2*** **and** **B** ***RORC2v1*** **in ICLN of paratuberculosis-diseased and uninfected control sheep.** Each point is the mean ΔCq of three biological replicates per animal, each in duplicate. ANOVA: **A** *RORC2*, *P* = 0.0004; **B** *RORC2v1*, *P* = 0.0026; error bars, ± SD. *≤0.05, >0.01; **≤0.01, 0.001; ***≤0.001.

Quantification of two *RORC2* variants showed overall significant differences (Table 2) for both the full length transcript (*RORC2, P* = 0.0004) and the truncated transcript (*RORC2v1, P* = 0.0026). Both transcripts behaved similarly in the between-group comparisons. *RORC2* and *RORC2v1* (Figure 2) showed significant 2.4 and 3.09 (both *P* ≤ 0.05) fold changes respectively in the M vs. C comparison; and significant 3.48 (*P* ≤ 0.001) and 3.49 (*P* ≤ 0.01) fold changes respectively in the *P* vs. C comparison. Neither was significantly different in the M vs. *P* comparison.

### *RORA* expression

**Figure 3:**
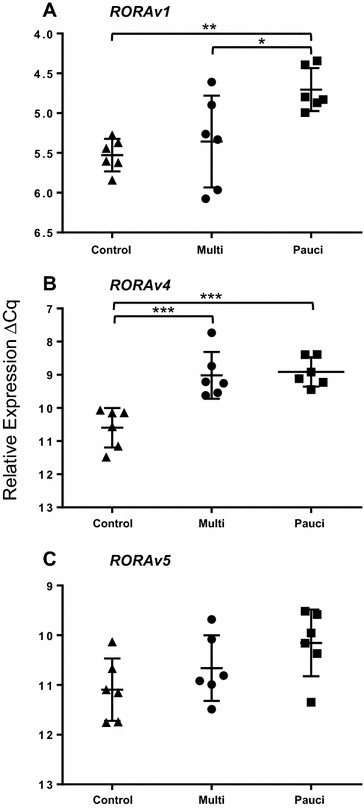
**Relative quantification of** **A** ***RORAv1***, **B** ***RORAv4*** **and** **C** ***RORAv5*** **in ICLN of paratuberculosis-diseased and uninfected control sheep.** Each point is the mean ΔCq of three biological replicates per animal, each in duplicate. ANOVA: **A** *RORAv1*, *P* = 0.0053; **B** *RORAv4*, *P* = 0.0002; **C** *RORAv5*, *P* = 0.07; error bars, ± SD. *≤0.05, >0.01; **≤0.01, 0.001; ***≤0.001.

Quantification of the *RORA* transcript variants could be performed only for *RORAv1*, *RORAv4* and *RORAv5* because expression levels of *RORAv2* and *RORAv3* were too low for accurate measurements (detected at >30 cycles, below the linear part of the titration curve). Overall significant differences (Table 2) were identified for both *RORAv1* (*P* = 0.0053) and *RORAv4* (*P* = 0.0002). *RORAv5* was marginally insignificant (*P* = 0.07). Comparison between the groups (Figure 3) showed that *RORAv1* was significantly increased in lymph nodes from paucibacillary animals (*P* vs. C; 2.49 fold, *P* ≤ 0.01 and (M vs. *P*; −1.9 fold, *P* ≤ 0.05). *RORAv4* was significantly increased in both the M vs. C (2.78 fold, *P* ≤ 0.001) and *P* vs. C comparisons (2.89 fold, *P* ≤ 0.001), but was equally expressed in the multi- and paucibacillary groups (−1.04 fold). *RORVv5* was not significantly different in any of the three comparisons, although it was increased by 2.67 fold in the *P* vs. C comparison.

## Discussion

Previous studies investigating cytokine transcripts indicated that sheep paucibacillary disease is strongly associated with Th1/Th17 activation and that multibacillary pathology is linked to a Th2 T cell response [7, 34, 35]. Tbx21, GATA-3, RORγt and RORα are the master regulator transcription factors responsible for controlling the polarization of these T cell subsets; and splice variants of these genes have been described in sheep, some of which are differentially expressed in gastrointestinal parasitic disease [31]. This study examined the relationship between the expression of the different variants of these T cell master regulators and the two paratuberculosis disease pathologies in naturally-infected sheep.

Only one *TBX21* transcript has been described in sheep, which showed significant increased expression in ICLN from paucibacillary animals when compared to both uninfected controls (2.6 fold, *P* ≤ 0.01) and multibacillary sheep (1.88 fold, *P* ≤ 0.05). These data contrast with measurements made from jejunal lymph nodes in experimentally-infected red deer [36], where there was no differential expression of *TBX21*, and *IFNG* levels were increased only in “severe disease” (multibacillary) cases. The difference might be associated with anatomical location of the lymph nodes, as Begg et al. [37] showed that responses to infection varied with their position. The immune response to paratuberculosis is greatest at the location of the most severe lesions [37]; the terminal ileum is a major site of the paratuberculosis lesions in sheep [3] and the ICLN is the major immune inductive site for that tissue. Tbx21 is required for Th1 activation as it induces the expression of IFNγ [18]; which is the critical cytokine for controlling intracellular mycobacterial replication [6]. High levels of both *TBX21* and *IFNG* transcripts are seen in human Crohn’s disease [38] and *TBX21* SNPs are linked with resistance to human pulmonary tuberculosis [39]. In sheep paratuberculosis therefore, high levels of *TBX21* expression may explain the increased levels of IFNγ [7, 40] and the consequent low numbers of MAP in paucibacillary lesions.

The expression of the full length *GATA3* variant was significantly increased (2.08 fold, *P* ≤ 0.05) in the paucibacillary sheep in comparison to multibacillary animals but was not significantly different in either of the diseased vs. control comparisons. GATA-3 is required for Th2 differentiation as it transactivates the *IL4* cassette and promotes the transcription of *IL4*, *IL5* and *IL13* [41]; it also inhibits Th1 responses and is repressed during Th1 development [42]. The increased levels of *GATA3* transcripts in paucibacillary sheep, with (non-significantly) reduced levels in multibacillary sheep (−1.6 fold) is similar to that found in red deer with high levels of *GATA3* (and *IL4*) in “minimal disease” (paucibacillary) and reduced expression in “severe disease” [36]; and these authors suggested that Th2 responses act to control immunopathology in infected animals, and that loss of these responses leads to the multibacillary disease state. *GATA3v1* expression showed no significant changes in any of the three comparisons. This variant, which is also found in humans, encodes for GATA-3 with a deletion at position 260 (glutamic acid). The deletion is not within the transactivation domains or either of the zinc-finger domains, nor is it associated with the conserved YxKxHxxxRP motif at position 345–354 that seems to control Th2 cytokine production [43]. To date this is the only study that describes any biological difference between full length *GATA3* and *GATA3v1* in any species.

Full length *RORC2* expression was significantly increased in both the *P* vs. C (3.48 fold, *P* ≤ 0.001) and M vs. C (2.4 fold, *P* ≤ 0.05) comparisons. *RORC2v1* behaved in an almost identical manner. Increased RORγt expression in humans is associated with a range of inflammatory pathologies, including inflammatory bowel disease [44]; and these data imply that RORγt-mediated Th17 activity is an important aspect of the chronic inflammation seen in both pathological forms of paratuberculosis. However, the 1.45 fold increase of *RORC2* in the *P* vs. M comparison (whilst not significant) indicates a trend of increased Th17 cell function in the paucibacillary immunopathology in comparison to multibacillary disease. The twelve amino acid deletion encoded by the *RORC2v1* variant is within the predicted ligand-binding domain [45], which implies a modification in function; a similar *RORC2* variant in humans suppresses *IL17A* and *IL21* transcription and consequently inhibits Th17 function [29]. However, there is no indication in these studies that the two sheep variants have any different functions.

Only three of the five *RORA* variants showed measurable levels of expression. *RORAv1* was significantly increased in paucibacillary sheep in comparison to both control (2.49 fold, *P* ≤ 0.01) and multibacillary sheep (1.9 fold, *P* ≤ 0.05); and *RORAv4* was increased in both the M vs. C (2.78 fold, *P* ≤ 0.001) and *P* vs. C comparisons (2.89 fold, *P* ≤ 0.001). *RORAv5* showed no significant differential expression but was 2.67 fold increased in the *P* vs. C comparison (but not significant). The biological activities of RORα are mediated by both the ligand-binding and DNA-binding domains [30]. In addition, the amino terminal A and B domains play important roles in intracellular localization, binding specificity and cell tropism. *RORAv1* and *RORAv5* possesses both ligand- and DNA- binding domains but different A and B domains [31]; *RORAv4,* which encodes the same protein as *RORAv2*, possesses the conserved ligand-binding domain but lacks the A, B and DNA-binding domains.

Optimal Th17 differentiation requires the co-expression of both RORγt and RORα [21] and the significantly high levels of *RORAv1* in paucibacillary (in comparison to both multibacillary and control sheep) further emphasises an important role for Th17 activation in paucibacillary (tuberculoid) pathology. It is possible that *RORAv4*, which is found in high levels in both paucibacillary and multibacillary sheep, could compete with *RORAv1* for ligand binding and inhibit its function, most effectively in those animals with lower levels of *RORAv1* (i.e. multibacillary), with consequent reduction in Th17 activation. This is similar to the differential expression patterns of *RORAv2* and *RORAv5* seen in parasitic disease pathologies [31].

In conclusion, this study measures the expression of the transcript variants of the master regulator transcription factors that control Th1, Th2 and Th17 T cell differentiation, in sheep with defined paucibacillary and multibacillary paratuberculosis pathology. Relatively low levels of *GATA3* in multibacillary animals confirms the data from red deer, but does not confirm that multibacillary (lepromatous) paratuberculosis is primarily caused by Th2 activation. High levels of expression of *TBX21*, *RORC2* and *RORC2v1* highlights the role of both Th1 and Th17 in controlling bacterial replication in paucibacillary disease, and increased expression of *RORAv1* emphasises that RORα plays an important part in Th17 development in this disease. The differentiation between paucibacillary and multibacillary pathology might also concern *RORAv4*, which may inhibit Th17 activation in multibacillary disease and further contribute to T cell dysfunction.

## Additional files

10.1186/s13567-016-0368-3 **Primer sets for RT-qPCR.** The primer sequences, their Tm (°C) and PCR product sizes used for RT-qPCR.
10.1186/s13567-016-0368-3 **Histopathology of lymph node of diseased animals.** Haematoxylin and eosin (H&E) and Ziehl-Neelsen (ZN) histopathology of lymph node from multibacillary (SH139) and paucibacillary (SH155) diseased animals. Inserts are terminal ileum from the same animals. All images are x10 magnification.
10.1186/s13567-016-0368-3 **5’ nucleotide sequences of** ***Ovis aries*** **T cell transcription factor variants.** A. *GATA3* (LN848231 and LN848232). B. *RORC2* (LN848233 and LN848234. C. *RORA* (LN848235, LN848236, LN848237, LN848238, LN848239).
