# Supplementary material for: Variations in T cell transcription factor gene structure and expression associated with the two disease forms of sheep paratuberculosis
Source: Vet Res. 2016 Aug 17;47:83. doi: 10.1186/s13567-016-0368-3 (PMC4988036; doi:10.1186/s13567-016-0368-3)
Supplement: Supplementary file 1 — 10.1186/s13567-016-0368-3 Primer sets for RT-qPCR. The primer sequences, their Tm (°C) and PCR product sizes used for RT-qPCR. [file 13567_2016_368_MOESM1_ESM.pdf]

Additional File 1 Primer sets for RT-qPCR

Gene	Primer sequences	Tm °C	Product size (bp)
TBX21	F: CCTGTTGTGGTCCAAGTTC<br>R: CGGTAATGGCTGGTGGGCTC	60	120
GATA3	F: CCACAAGATGAACGGACAG<br>R: GGCATTTCTTCTCCACAGAGTCGT	62	129
GATA3v1	F: GCGAGATCCAGCACAGGCC<br>R: GTTCTGTCCGTTCATCTTGTGG	60	131
RORC2	F: CGCTGTGCCCACCGACTCACCGAG<br>R: TGACCAGCACCACTTCCATG	62	136
RORC2v1	F: TTGAAGGCTGCAGTGAACTC<br>R: GGCATTGATGAGCACGAGG	63	116
RORAv1	F: GTGATCGCAGCGATGAAAGC<br>R: CCTTGCAGCCTTCACATGTAATG	60	101
RORAv2	F: AGTTCTCTGCTGCAGTTGCTAAC<br>R: GACGAGCTCATGGGCAAGG	62	124
RORAv3	F: TGCGCAGACAGAGCTATTCC<br>R: TGGGTCTTCTTTGTTACTGAGATACC	62	112
RORAv4	F: GCAGCTTTCTTCTGGTGTCGTC<br>R: TGGGTCTTCTTTGTTACTGAGATACC	60	113
RORAv5	F: GGCTTTCTGTGGATGGGATC<br>R: TGGGTCTTCTTTGTTACTGAGATACC	60	133
YWHAZ	F: TGTAGGAGCCCGTAGGTCATC<br>R: TCTCTCTGTATTCTCGAGCCATC	60	101
SDHA	F: ACCTGATGCTTTGTGCTCTGC<br>R: CCTGGATGGGCTTGGAGTAA	62	126
