# Supplementary material for: Variations in T cell transcription factor gene structure and expression associated with the two disease forms of sheep paratuberculosis
Source: Vet Res. 2016 Aug 17;47:83. doi: 10.1186/s13567-016-0368-3 (PMC4988036; doi:10.1186/s13567-016-0368-3)

Multibacillary paratuberculosis

ileo-caecal lymph node x10

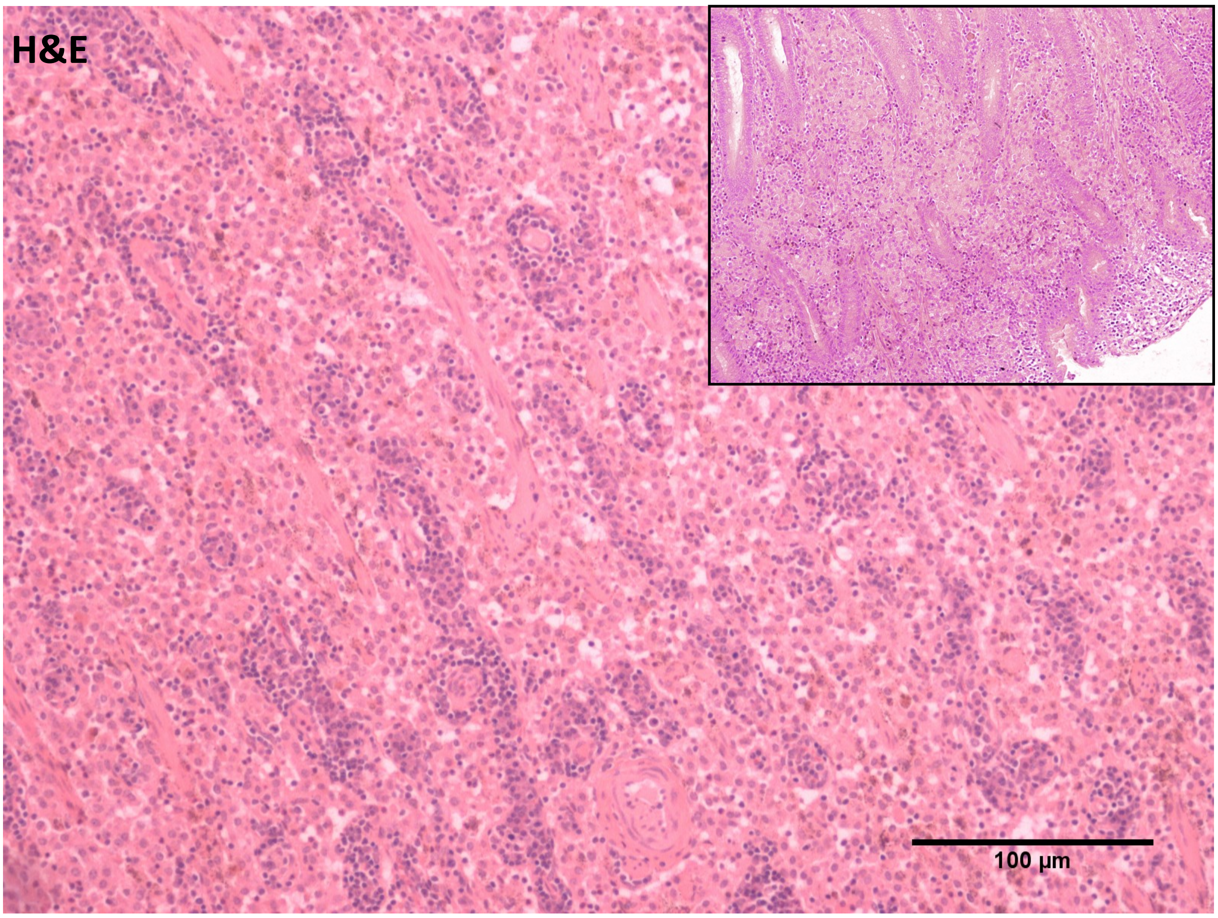

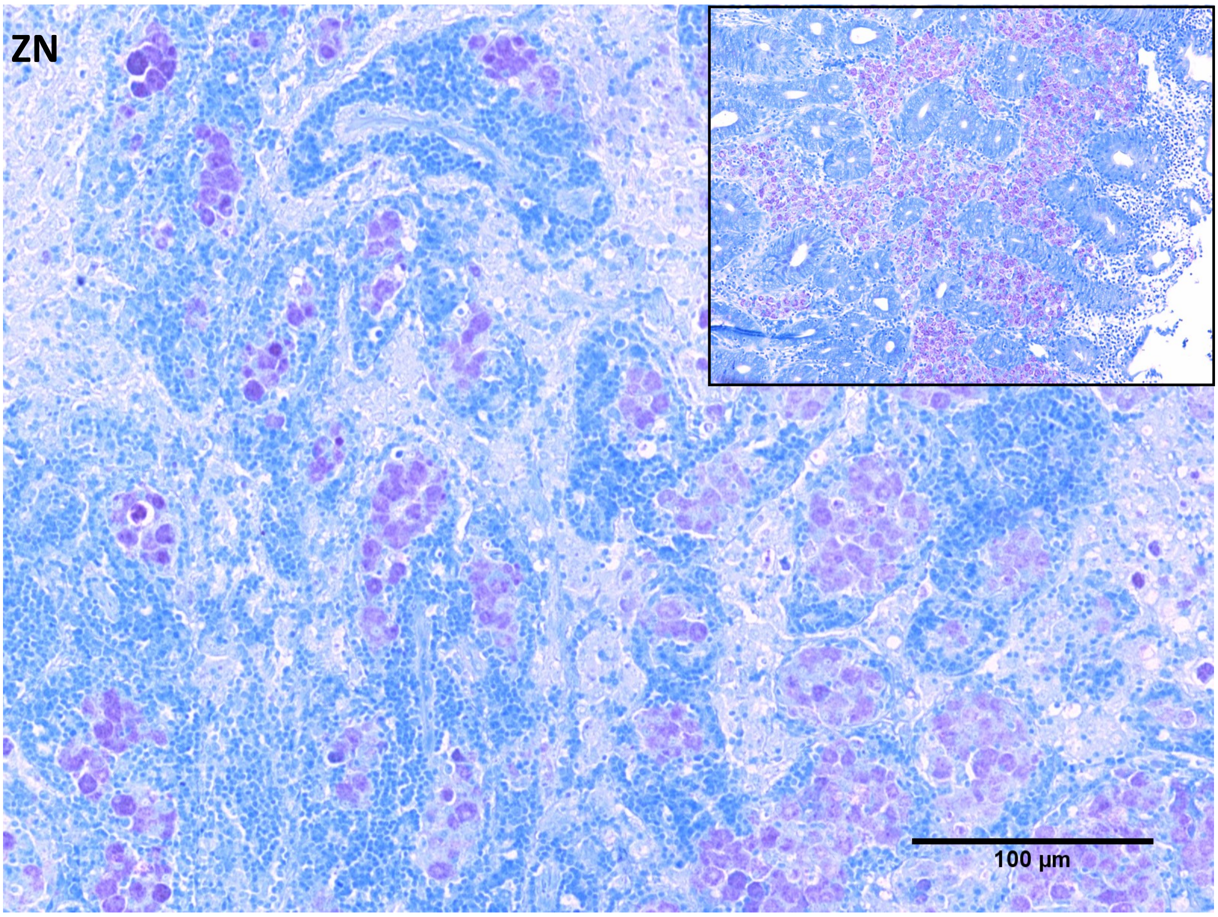

Paucibacillary paratuberculosis

ileo-caecal lymph node x10

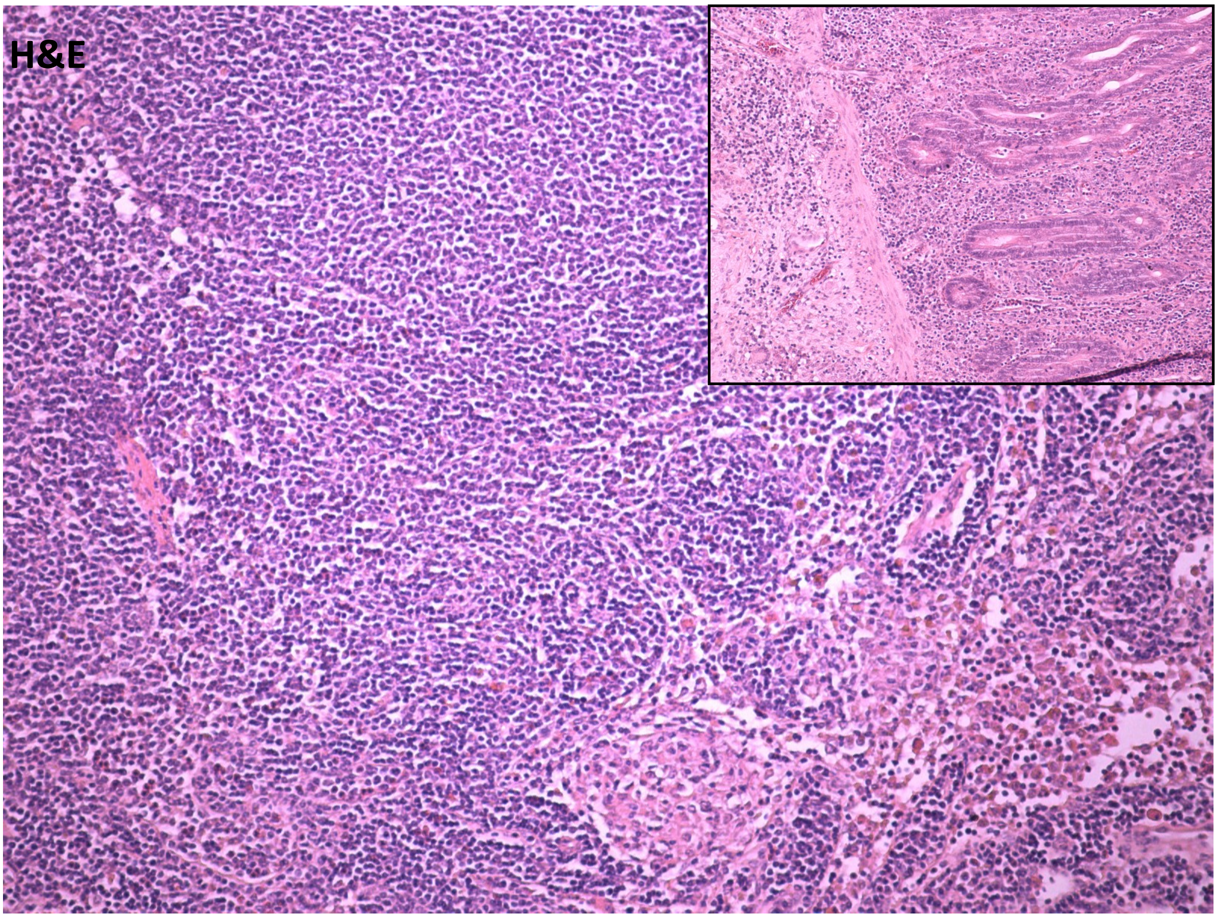

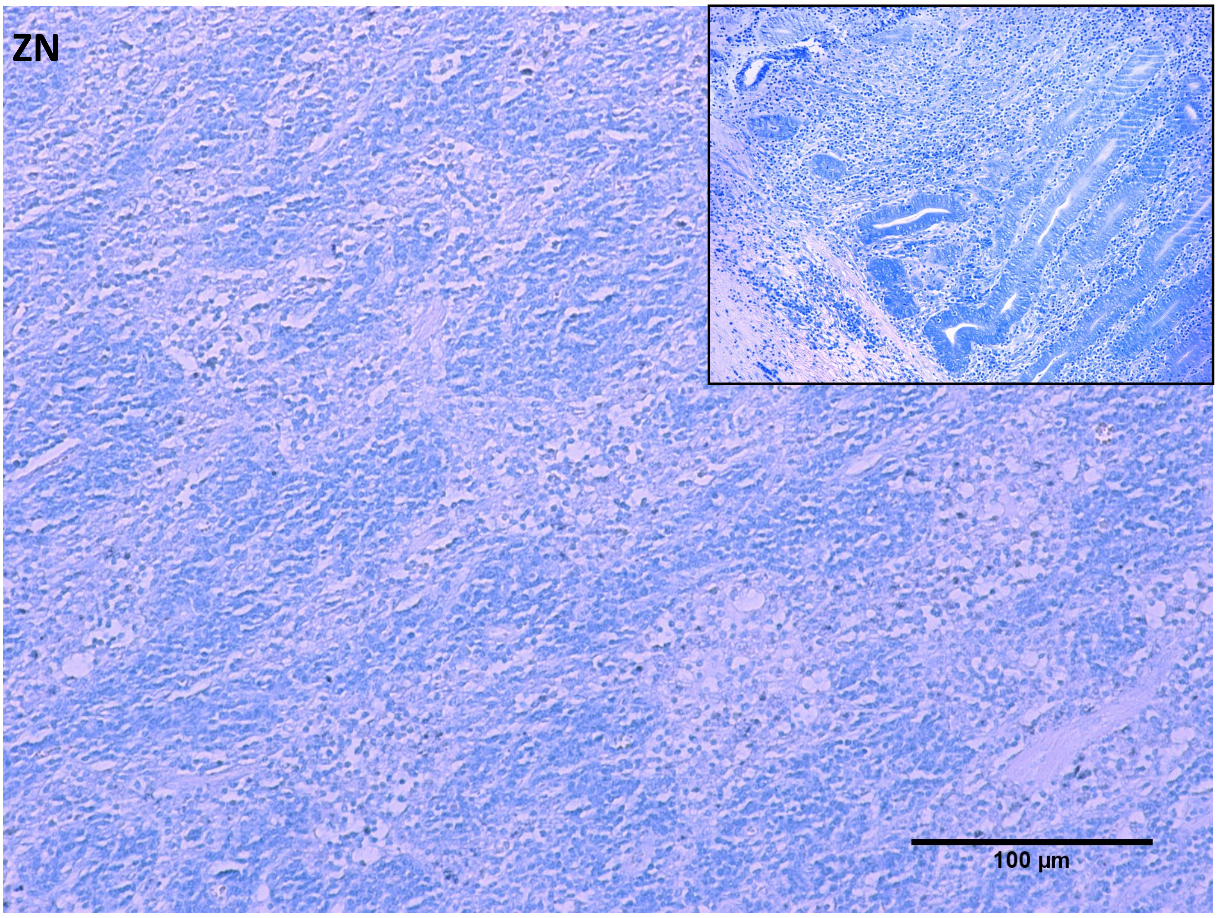

Supplement: Supplementary file 2 — 10.1186/s13567-016-0368-3 Histopathology of lymph node of diseased animals. Haematoxylin and eosin (H&E) and Ziehl-Neelsen (ZN) histopathology of lymph node from multibacillary (SH139) and paucibacillary (SH155) diseased animals. Inserts are terminal ileum from the same animals. All images are x10 magnification. [file 13567_2016_368_MOESM2_ESM.pdf]
