# Supplementary material for: Variations in T cell transcription factor gene structure and expression associated with the two disease forms of sheep paratuberculosis
Source: Vet Res. 2016 Aug 17;47:83. doi: 10.1186/s13567-016-0368-3 (PMC4988036; doi:10.1186/s13567-016-0368-3)
Supplement: Supplementary file 3 — 10.1186/s13567-016-0368-3 5’ nucleotide sequences of Ovis aries T cell transcription factor variants. A. GATA3 (LN848231 and LN848232). B. RORC2 (LN848233 and LN848234. C. RORA (LN848235, LN848236, LN848237, LN848238, LN848239). [file 13567_2016_368_MOESM3_ESM.pdf]

Additional File 3 5’ nucleotide sequences of *Ovis aries* T cell transcription factor variants

A. *GATA3* (LN848231 and LN848232)

```
GATA3       GAGGCCCAAGGCGAGATCCAGCACAGAAGGCCGGGAGTGTGTGAACTGCGGGGCGACATC   840
GATA3v1     GAGGCCCAAGGCGAGATCCAGCACA---GGCCGGGAGTGTGTGAACTGCGGGGCGACATC   837
            *************************   ********************************
```

B. *RORC2* (LN848233 and LN848234)

```
RORC2       CCTACAACGCTGACAACAACACAGTCTTTTTTGAAGGCAAATACGGTGGCGTGGAGCTGT  1260
RORC2v1     CCTACAACGCTGACAACAACACAGTCTTTTTTGAAG------------------------  1236
            ************************************

RORC2       TCCGAGCCTTGGGCTGCAGTGAACTCATCAGCTCCATCTTTGACTTCTCCCGCTCCCTGA  1320
ROR2Cv1     ------------GCTGCAGTGAACTCATCAGCTCCATCTTTGACTTCTCCCGCTCCCTGA  1284
                        ************************************************
```

C. *RORA* (LN848235, LN848236, LN848237, LN848238, LN848239)

```
RORAv1      ------------------------------------------------------------   0
RORAv2      CGATTCCTCGGGGTTTCACAACCTAAGAGGTCTGGGAGGGCACCTTGCAGTGCCTAAGTC   60
RORAv3      ------------------------------------------------------------   0
RORAv4      ------------------------------------------------------------   0
RORAv5      CGATTCCTCGGGGTTTCACAACCTAAGAGGTCTGGGAGGGCACCTTGCAGTGCCTAAGTC   60

RORAv1      ------------------------------------------------------------   0
RORAv2      TCCACGAGTGTTCAGAGCGGACATAAAATGTACAGAGTCTTTAGACAAGCGG---GCTTT   117
RORAv3      -------------TGCGCAGA---------------------------------------   8
RORAv4      --------------ACACTGACATGGACTGAA-GGAG----TAGAAAAGAAGGCAGCTTT   41
RORAv5      TCCACGAGTGTTCAGAGCGGACATAAAATGTACGGAGTCTTTAGACAAGCGG---GCTTT   117

RORAv1      ------------------------------------------------------------   0
RORAv2      CTGTGGATGGGATCCGCCTCTGGGAGGCCAGGG-AAAAGACAAAAACAAGTGTTTCCTGC   176
RORAv3      CAGAGCT---ATTCCAGCACCAG-----CAGAGGG---------------TTATCCATGC   45
RORAv4      CTTCTGGTGTCGTCAGCCTCTTGCAT--CTGGGTCC--------------AGGTCCCTTC   85
RORAv5      CTGTGGATGGGATCCGCCTCTGGGAGGCCAGGGGAAAAGACAAAAACAAGTGTTTCCTGC   177

RORAv1      ------------------------------------------------------------   0
RORAv2      AGTTCTCTGCTGCAGTTGCTAACAGAGAGT---CACTCGACGCTTGGTATCTCAGTAACA   233
RORAv3      CAGTGTCCAGTGAGG----GAACTGAGAGAAGGCGGCATGATCCAGGTATCTCAGTAACA   101
RORAv4      AAGGTTAATCA---G----AATCAGAA---GTTCAGAGAGCTGCAGGTATCTCAGTAACA   135
RORAv5      AGTTCTCTGCTGCAGTTGCTAACAGAGAGT---CACTCGACGCTTGGTATCTCAGTAACA   234

RORAv1      -------------------------------------------TCACCGCG---------   8
RORAv2      AAGAAGACCCATACATGGCAGGCATGGTACAGAGAGCTTCCTTGCACAGCCTTGCCCATG   293
RORAv3      AAGAAGACCCATACAT--------------------------------------------   117
RORAv4      AAGAAGACCCATACAT--------------------------------------------   151
RORAv5      AAGAAGACCCATACAT--------------------------------------------   250

RORAv1      -GCTTAAATGATGTATTTTGTGATCGCAGCGATGAAAGCTCAAATTGAAATTATTCCATG   67
RORAv2      AGCTCGTCCCTGGCCTGGTGGGACCCACTCTAGAGGTGCTCAAATTGAAATTATTCCATG   353
RORAv3      ---------------------------------------CTCAAATTGAAATTATTCCATG   139
RORAv4      ---------------------------------------CTCAAATTGAAATTATTCCATG   173
RORAv5      ---------------------------------------CTCAAATTGAAATTATTCCATG   272
                                                   *********************
```

RORAv1	**CAAGATCTGTGGAGACAAATCATCAGGAATCCATTACGGTGTCATTACATGTGAAGGCTG**	127
RORAv2	CAAGATCTGTGGAGACAAATCATCAGGAATCCATTACGGTGTCATTACATGTGAAGGCTG	413
RORAv3	**CAAGATCTGTGGAGACAAATCATCAGGAATCCATTACGGTGTCATTACATGTGAAGGCTG**	199
RORAv4	CAAGATCTGTGGAGACAAATCATCAGGAATCCATTACGGTGTCATTACATGTGAAGGCTG	233
RORAv5	**CAAGATCTGTGGAGACAAATCATCAGGAATCCATTACGGTGTCATTACATGTGAAGGCTG**	332
	**	
RORAv1	**CAAGGGCTTTTTCAGGAGAAGTCAGCAAAGCAATGCCACCTACTCCTGTCCTCGTCAAAA**	187
RORAv2	CAAGGGCTTTTTCAGGAGAAGTCAGCAAAGCAATGCCACCTACTCCTGTCCTCGTCAAAA	473
RORAv3	**CAAGGGCTTTTTCAGGAGAAGTCAGCAAAGCAATGCCACCTACTCCTGTCCTCGTCAAAA**	259
RORAv4	CAAGGGCTTTTTCAGGAGAAGTCAGCAAAGCAATGCCACCTACTCCTGTCCTCGTCAAAA	293
RORAv5	**CAAGGGCTTTTTCAGGAGAAGTCAGCAAAGCAATGCCACCTACTCCTGTCCTCGTCAAAA**	392
	**	
RORAv1	**GAACTGTTTGATTGATCGGACCAGTAGAAACCGCTGCCAGCACTGTCGATTACAGAAATG**	247
RORAv2	GAACTGTTTGATTGATCGGACCAGTAGAAACCGCTGCCAGCACTGTCGATTACAGAAATG	533
RORAv3	**GAACTGTTTGATTGATCGGGCCAGTAGAAACCGCTGCCAGCACTGTCGATTACAGAAATG**	319
RORAv4	GAACTGTTTGATTGATCGGACCAGTAGAAACCGCTGCCAGCACTGTCGATTACAGAAATG	353
RORAv5	**GAACTGTTTGATTGATCGGACCAGTAGAAACCGCTGCCAGCACTGTCGATTACAGAAATG**	452
	*******************.**	
RORAv1	**CCTTGCCGTGGGGATGTCTCGAGATGCTGTGAAATTTGGCCGCATGTCGAAAAAGCAGAG**	307
RORAv2	CCTTGCCGTGGGG**ATGTCTCGAGATGCTGTGAAATTTGGCCGCATGTCGAAAAAGCAGAG**	593
RORAv3	**CCTTGCCGTGGGGATGTCTCGAGATGCTGTGAAATTTGGCCGCATGTCGAAAAAGCAGAG**	379
RORAv4	CCTTGCCGTGGGG**ATGTCTCGAGATGCTGTGAAATTTGGCCGCATGTCGAAAAAGCAGAG**	413
RORAv5	**CCTTGCCGTGGGGATGTCTCGAGATGCTGTGAAATTTGGCCGCATGTCGAAAAAGCAGAG**	512
	**	

Bold; coding region
